# Preparation and Characterization of Semi-Alicyclic Polyimides Containing Trifluoromethyl Groups for Optoelectronic Application

**DOI:** 10.3390/polym12071532

**Published:** 2020-07-11

**Authors:** Mei Zhang, Weili Liu, Xia Gao, Peng Cui, Tao Zou, Guanghui Hu, Liming Tao, Lei Zhai

**Affiliations:** 1 Beijing Center for Physical and Chemical Analysis, Beijing 100094, China; liuweili@bcpca.ac.cn (W.L.); gaoxia@bcpca.ac.cn (X.G.); cuipeng@bcpca.ac.cn (P.C.); zoutao@bcpca.ac.cn (T.Z.); huguanghui@bcpca.ac.cn (G.H.); 2 Beijing Key Laboratory of Detection Technology and Quality Evaluation of Organic Material, Beijing 100094, China; 3 State Key Laboratory of Solid Lubrication, Lanzhou Institute of Chemical Physics, Chinese Academy of Sciences, Lanzhou 730000, China; 4 Laboratory of Advanced Polymer Materials, Institute of Chemistry, Chinese Academy of Sciences, Beijing 100190, China; zhailei@iccas.ac.cn

**Keywords:** polyimide, alicyclic structure, trifluoromethyl, transparency, structure–property relationship

## Abstract

Transparent polyimides (PI) films with outstanding overall performance are attractive for next generation optoelectronic and microelectronic applications. Semi-alicyclic PIs derived from alicyclic dianhydrides and aromatic diamines have proved effective to prepare transparent PIs with high transmittance. To optimize the combined properties of semi-alicyclic PIs, incorporating bulky trifluoromethyl groups into the backbones is regarded as a powerful tool. However, the lack of fundamental understanding of structure–property relationships of fluorinated semi-alicyclic PIs constrains the design and engineering of advanced films for such challenging applications. Herein, a series of semi-alicyclic PIs derived from alicyclic dianhydrides and trifluoromethyl-containing aromatic diamines was synthesized by solution polycondensation at high temperature. The effects of alicyclic structures and bulky trifluoromethyl groups on thermal, dielectric and optical properties of PIs were investigated systematically. These PI films had excellent solubility, low water absorption and good mechanical property. They showed high heat resistance with *T*_g_ in the range of 294–390 °C. It is noted that tensile strength and thermal stability were greatly affected by the rigid linkages and alicyclic moieties, respectively. These films exhibited obviously low refractive indices and significantly reduced dielectric constants from 2.61 to 2.76, together with low optical birefringence and dielectric anisotropy. Highly transparent films exhibited cutoff wavelength even as low as 298 nm and transmittance at 500 nm over 85%, displaying almost colorless appearance with yellowness index (b*) below 4.2. The remarkable optical improvement should be mainly ascribed to both weak electron-accepting alicyclic units and bulky electron-withdrawing trifluoromethyl or sulfone groups. The present work provides an effective strategy to design molecular structures of optically transparent PIs for a trade-off between solution-processability, low water uptake, good toughness, high heat resistance, low dielectric constant and excellent optical transparency.

## 1. Introduction

With the development of electronics, microelectronics and large-scale integrated circuits, flexible substrates have attracted considerable interest due to their promising applications in next-generation displays, diverse flexible electronic devices and aerospace industry [1,2,3]. The plastic substrates have been tried to build light-weight and conveniently portable devices, foldable phones or roll-up displays, flexible printed circuit board. As one of key components in devices, the plastic substrate has great influence on the performance and reliability of devices. Whereas traditional organic polymer materials are difficult to meet the required demands of flexible substrates. There is an urgent need for high-performance polymers available for the advanced manufacturing. Polyimides (PI) are a class of engineering plastics widely applied in electronics, microelectronics, aerospace and energy fields, due to their outstanding thermal, mechanical, chemical resistance and electrical properties [4,5,6,7,8]. Thus, PIs are regarded as the best choice for substrate materials used for flexible optoelectronic applications such as organic electroluminescent displays, flexible thin film solar cells, flexible printed circuits boards with multilayers, etc [9,10,11,12]. However, it is difficult for conventional PIs with fully-aromatic repeating units to meet the harsh requirements when applied for different situations. Most of PIs have so poor solution processability in common organic solvents that their films can be only prepared by thermal imidization using the precursor solutions. In addition, fully-aromatic PI films are well known to show yellow or brown coloration and high dielectric constant, which are unsuitable for various applications required optical transparency or low permittivity [13,14]. These obstacles greatly limit the widespread use of PIs in the advanced optoelectronic and microelectronics fields. Therefore, developing PIs with good processability while simultaneously maintaining outstanding overall performance such as high heat resistance, excellent optical transparency and low dielectric features is still a great challenge.

For fully-aromatic PIs, their intense coloration and high absorption in the ultraviolet-visible region have proved to be a consequence of easily formed charge transfer complexes (CTC) in their highly conjugated molecular chains. Ando and coworkers have systematically investigated the relationship between molecular structures and coloration of PIs [15,16,17]. In general, the electron-deficient dianhydride units and electron-rich diamine units act as the electron donor and acceptor, respectively. The charge interactions between them are the main reason for the formation of inter- and/or intra-molecular CTC. It is further reported that optical property of PIs is dominated by the electron-donation abilities of diamines when the dianhydride structure is fixed. In order to obtain transparent or even colorless PIs, various effective strategies have been reported to suppress or eliminate the charge transfer interactions. Researchers, respectively, introduce bulky pendant substituents, flexible linkages, noncoplanar or asymmetric units, non-aromatic structures or electron-withdrawing functional groups into polymer backbone [18,19,20,21]. Among these strategies, the adoption of alicyclic dianhydrides with weak electron-accepting abilities or alicyclic/aliphatic diamines with weak electron-donating abilities can effectively suppress the formation of CTC. Those PIs with non-aromatic units exhibit high transparency and meantime low coloration compared to fully-aromatic PIs. Especially, semi-alicyclic PIs derived from alicyclic dianhydrides and aromatic diamines are taken as the best choice to obtain transparent PIs with high transmittance because their reactions can proceed smoothly without salt formation [22,23,24,25]. In the previous work [26], semi-alicyclic PIs based on 1,2,4,5-cyclohexanetetracarboxylic dianhydride (H-PMDA, also named CHDA) and various aromatic diamines were prepared and showed considerably improved optical transparency with entirely colorless appearance. Whereas, it is also found the steric structures of H-PMDA have a significant influence on the polymerizability and thermal expansion of resultant PIs. 

As is well known, high molecular weight of semi-alicyclic PIs is very important because it is directly connected to the ductility of resulting film. However, most cycloaliphatic tetracarboxylic dianhydrides do not have sufficient reactivity with aromatic diamines, and they have also problems of low-cost mass productivity. As a representative alicyclic dianhydride, H-PMDA does not have sufficient polymerizability due to its steric structures containing three isomers. Hasegawa systematically investigated the steric effect on reactivity of isomers, including two boat-forms and one chair-from [11]. For commercially available H-PMDA product, it contains three isomers with different proportions that are difficult to separate and thus greatly limit its polymerizability. More importantly, the steric structures also have a major impact on the extended PI chain forms. It has been found that these semi-alicyclic PIs based on H-PMDA with mixed isomers generally have inferior thermal dimensional stability. Furthermore, in theory, alicyclic units in the molecular structures can also contribute to the improved solubility owing to the decreased content of conjugated backbone and weakened molecular interaction. It has been already found that alicyclic dianhydrides can remarkably improve optical transparency, but it may be not helpful to increase the solubility as expected if the diamine structural design is not reasonable. For example, semi-alicyclic PI derived from 1,2,3,4-cyclobutanetetracarboxylic dianhydride (CBDA) and 2,2’-bis(trifluoromethyl)-4,4’-diamino biphenyl (TFMB) can obtain outstanding optical properties and relatively low thermal expansion coefficients (CTE) around 21 ppm/°C [27,28]. However, this PI is not compatible with chemical imidization method and cannot be processed by common organic solvents even if it has a high fluorine content. CBDA-based PIs also tend to result in other undesirable properties. Furthermore, Liu and coworkers prepared fluorinated semi-alicyclic transparent PI films for optocommunication applications, which were based on CBDA and aromatic diamines including 1,4-bis(4-amino-2-trifluoromethyl-phenoxy) benzene (6FAPB) and 4,4’-bis(4-amino-2-trifluoromethylphenoxy) biphenyl (6FBAB) [29]. However, these CBDA-based PIs showed much low heat resistance with glass transition temperatures (*T*_g_) below 245 °C. In brief, determining how to effectively balance optical transmittance and other desired properties is very crucial for those semi-alicyclic PIs using alicyclic dianhydrides and aromatic diamines.

A large number of studies have shown that semi-alicyclic PIs, together with modification of aromatic diamine moieties, can achieve high combined performance without sacrificing optical properties. It is reported that these transparent PIs showed excellent solubility and low dielectric constants when bulky electron-withdrawing trifluoromethyl (–CF_3_) and sulfonyl groups (–SO_2_–) were incorporated in diamine moieties [11]. Besides, in order to improve chemical resistance of transparent films, semi-alicyclic PIs based on bicycle [2.2.2]oct-7-ene-2,3,5,6-tetracarboxylic dianhydride (BODA) and aromatic diamines containing *ortho*-substituted groups were developed [26]. It is found that these semi-alicyclic PIs with fluorine content lower than 10 wt% (by introducing fluorine atoms (-F)) have outstanding solubility and high heat resistance, but they also show inferior toughness and relatively higher yellow indices. These results indicated that incorporating trifluoromethyl groups into diamine moieties is a more effective design strategy to improve the transparency and coloration of semi-alicyclic PIs. Moreover, in terms of molecular design of intrinsic low dielectric PIs, increasing inherent free volume or incorporating groups with low electronic polarization was proved to be effective to reduce dielectric constant [30,31,32]. Bulky trifluoromethyl groups undoubtedly increased free volume in the molecular structures that can dilute the polar molecular concentration, and weaken electronic interaction between polymer chains [33,34]. This means incorporating bulky trifluoromethyl groups into backbones provides a powerful tool to optimize the combined properties of semi-alicyclic PIs. Many different semi-alicyclic PI systems have been developed, however, the structure–property relationship of CF_3_-containing semi-alicyclic PIs is not completely clear, and the trade-off between solution-processability, water uptake, toughness, heat resistance, dielectric property and optical transparency needs further elaboration.

In the present study, we prepared a series of semi-alicyclic PIs with different rigid or flexible linkages by solution polycondensation at high temperature, using two alicyclic dianhydrides and five aromatic diamines containing trifluoromethyl groups. In view of the effect of steric structures on polymerizability and properties, cycloaliphatic dianhydride of H-PMDA containing three isomers was not used. Instead, BODA and CBDA with good chemical reactivity and cost advantage of mass production were adopted as the representative alicyclic dianhydrides. Especially, cycloaliphatic BODA, without the problem of different isomer reactivity has been proved to have good reactivity with aromatic diamines, and the resultant BODA-based PIs also exhibited excellent optical transparency and high *T*_g_ values [26]. Lu and other researchers also obtained similar results by using BODA and other aromatic diamine with bulky pendant groups [35]. *T*_g_ value of reported BODA-based PI was determined to exceed 420 °C, and this transparent film showed high transmittance over 86% in the visible region. In addition, considering that cycloaliphatic PIs often yield brittle films that cannot meet the toughness requirement for practical use, we introduce flexible ether bonds (–O–) into diamines to enhance flexibility except the biphenyl-based TFMB. Considering the poor solubility of PI derived from CBDA and TFMB, diamine containing trifluoromethyl and sulfonyl groups i.e., 2,2’-bis[4-(4-amino-2-trifluoromethyl-phenoxy) phenyl]sulfone (6FBAS) was chosen to polymerize with CBDA in order to ensure both the solubility and transparency of PI film. In the previous work, the sulfonyl and trifluoromethyl groups in the 6FBAS have been demonstrated to result in highly distorted molecular chains and strong electron-withdrawing effect, which can effectively suppress the formation of inter- and intra-molecular CTC [36,37]. Furthermore, the nonconjugated alicyclic structures and bulky electron-withdrawing trifluoromethyl groups are simultaneously introduced into backbones to inhibit charge transfer interactions. The synergistic effects of alicyclic structures and fluorinated groups on the performance of semi-alicyclic PIs, together with solubility, mechanical, thermal, dielectric, optical transparency and other properties were investigated systematically. Their aggregation of polymer chains was also evaluated by Wide angle X–ray diffractometry, and molecular orbital energies of model compounds were determined by density functional theory (DFT) calculations to clarify the effects of molecular structures on charge transfer interactions. Fundamental insights into structure–property relationships of semi-alicyclic PIs containing trifluoromethyl groups observed in this study offer guidance for the design of flexible transparent PIs for next-generation optoelectronic applications.

## 2. Materials and Methods

### 2.1. Materials

2,2’-Bis(trifluoromethyl)-4,4’-diamino biphenyl (TFMB), bicyclo[2.2.2]oct-7-ene-2,3,5,6- tetracarboxylic dianhydride (BODA) and 1,2,3,4-cyclobutanetetracarboxylic dianhydride (CBDA) were purchased from TCI Shanghai Co., Ltd. (Shanghai, China). 1,4-Bis(4-amino-2-trifluoromethyl-phenoxy) benzene (6FAPB) and 4,4’-bis(4-amino-2-trifluoromethylphenoxy) biphenyl (6FBAB) were purchased from Changzhou Sunlight Pharmaceutical Co., Ltd. (Changzhou, China). 2,2’-Bis[4-(4-amino-2-trifluoromethyl-phenoxy)phenyl]sulfone (6FBAS) was synthesized according to the literature [36,37]. All the diamines and dianhydrides were dried at 80–120 °C for 12 h under vacuum prior to use, respectively. Anhydrous *N*-methylpyrrolidone (NMP), isoquinoline and toluene were obtained from Sinopharm Chemical Reagent Co., Ltd. (Beijng, China). The solvent NMP was purified by distillation under reduced pressure before use, and other regents were used as received.

### 2.2. Characterization

The number and weight of the average molecular weights (*M*_n_ and *M*_w_) test were based on tandem gel permeation chromatography (GPC system, Waters, Milford, MA, USA) with solvent DMF containing 0.02 M LiBr as the eluent, detecting by multiangle laser light scattering system (Wyatt, Santa Barbara, CA, USA). The molecular weight distribution of polymers was calculated by *M*_w_/*M*_n_. Infrared spectroscopy was measured by a Fourier transform spectrophotometer (782, PerkinElmer, Waltham, MA, USA) using attenuated total reflection mode. ^1^H spectra were recorded on a nuclear magnetic resonance (NMR) spectrometer (DD2, Agilent, Santa Clara, CA, USA). Water absorption rate (*W*_A_) of PI films was measured according to the standard of GB/T 1034–2008, testing five specimens with dimension of 50 mm × 50 mm before immersion in the deionized water at 25 °C for 24 h. Static contact angle tests were performed with the optical contact angle system (OCA25, Dataphysics Instruments, Filderstadt, Germany) at room temperature. The surface of specimen was wiped with ethanol and dried before testing. Distilled water was used and at least three measurements were taken for each sample.

Tensile properties of films were measured by a universal tensile apparatus (3365, Instron, Norwood, MA, USA) with five specimens prepared according to the standard of GB/T 1040.3–2006 recommendations at a drawing rate of 5 mm/min. Thermal diffusivities measurements along the out-of-plane direction of PI films were conducted on a laser flash apparatus (LFA447, Netzsch, Selb, Germany). Each sample was around 50 μm and at least three specimens of each sample were examined. Glass transition temperature (*T*_g_) was obtained by dynamic mechanical analysis at a heating rate of 5 °C/min in nitrogen (242E, Netzsch, Selb, Germany), and the peak temperature of loss tangent (*tan δ*) curves was regarded as the *T*_g_ values. The film specimens (6 mm wide, 12 mm long, 50 μm thick) were measured with a frequency of 1 Hz and amplitude of 15 μm using tension mode. Thermal stability of PI films was evaluated by thermogravimetric analysis (STA449F5, Netzsch, Selb, Germany) with a heating rate of 10 °C/min under nitrogen. Linear thermal expansion coefficients (CTE) in the film plane were determined with thermomechanical analysis (DIL 402SE, Netzsch, Selb, Germany) at a heating rate of 5 °C/min in nitrogen. The average CTE values were calculated in the 50–200 °C range with data collected from the second heating.

The in-plane (*n*_∥_) and out-of-plane (*n*_⊥_) refractive indices measurements were performed on a prism coupler (2010, Metricon, Pennington, NJ, USA) at the wavelength of 632.8 nm. The average refractive index (*n_av_*) and birefringence (Δ*n*) were calculated by the equations *n_av_* = (2*n*_∥_ + *n*_⊥_)/3, ∆*n* = *n*_∥_ − *n*_⊥_. Optical dielectric constant (*ε*_opt_) of PI film was estimated from the empirical relationship with average refractive indices by Maxwell’s equation *ε*_opt_ = 1.1*n_av_*^2^. Anisotropy of in-plane and out-of-plane dielectric constant was defined as Δ*ε* = *ε*_∥_ − *ε*_⊥_ = 1.1(*n*_∥_^2^ − *n*_⊥_^2^). UV–Vis spectra of PI films was measured by the spectrophotometer (U3900, Hitachi, Tokyo, Japan) with samples thickness around 25 ± 3 μm. Color intensity of about 50 ± 3 μm thick films was evaluated by color spectrophotometer (i7, Xrite, Grand Rapids, MI, USA), and the color parameters were calculated according to the CIE LAB color difference equation. Wide angle X–ray diffractometry (WAXD) measurements was performed by a X–ray diffractometer (D/max 2500, Rigaku, Tokyo, Japan) using Cu/Kα radiation (*λ* = 1.54 Å) at room temperature. Density functional theory (DFT) with a three-parameter Becke-style hybrid functional (B3LYP) was used to calculate the molecular orbital energies for model compounds of semi-alicyclic PIs, using the 6-311++G(d,p) basis set. The highest occupied molecular orbital energy (*E*_HOMO_) and the lowest unoccupied molecular orbital energy (*E*_LUMO_) were obtained, and their difference energies i.e., energy band gap (*E*_GAP_) were calculated by the equation *E*_GAP_ = *E*_LUMO_ − *E*_HOMO_.

### 2.3. Synthesis of Semi-Alicyclic Polyimides and Preparation of Films

**Scheme 1:**
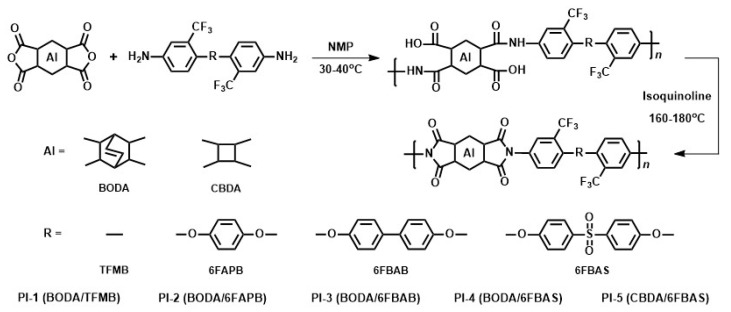
Synthesis of semi-alicyclic polyimides containing trifluoromethyl groups.

Semi-alicyclic PIs were prepared by solution polycondensation at high temperature using two alicyclic dianhydrides and four aromatic diamines containing trifluoromethyl groups, respectively. As shown in Scheme 1, poly(amic acid)s (PAA) as precursor solutions were firstly synthesized by polymerization of dianhydrides and diamines with equimolar amounts. PAA solutions were thermally cyclodehydrated at high temperatures to give corresponding semi-alicyclic PIs. As a typical experiment, PI-1 (BODA/TFMB) was synthesized and the film was accordingly prepared in the following procedure.

The dried diamine TFMB (32.02 g, 0.1 mol) was completely dissolved in anhydrous NMP (170 mL). Then, alicyclic dianhydride BODA (24.82 g, 0.1 mol) and isoquinoline (0.5 g) were added to the dissolved solution and stirred at 30–40 °C for 24 h under nitrogen. The homogeneous PAA solution was obtained and followed by imidization procedure. Toluene (35 mL) was added, and the reaction solution was gradually heated to 160–180 °C maintained for 24 h with continuous stirring. Water as a byproduct of polycondensation was appropriately removed by azeotropic distillation. Finally, the viscous solution was cooled down to room temperature and slowly poured into methanol to give a fiber-like precipitate. After filtration and drying, the PI precipitate was successfully obtained.

To prepare PI film, dried precipitate was dissolved in NMP with a solid content of 25 wt.%. The homogeneous PI solution was filtered and deaerated. Subsequently, the solution was uniformly casted on a clean glass plate by the automatic film applicator (4340, Elcometer, Manchester, UK), and thermally baked according to the elevated temperature procedure: 60 °C for 2 h, 150 °C for 1 h and 300 °C for 1 h, successively. Finally, robust PI film was obtained after it was immersed in water and peeled off from glass substrate. Other semi-alicyclic PI films, i.e., PI-2 (BODA/6FAPB), PI-3 (BODA/6FBAB), PI-4 (BODA/6FBAS) and PAI-5 (CBDA/6FBAS) were prepared according to the above procedure. The thickness of PI films was controlled around 25 ± 3 μm or 50 ± 3 μm for different specific measurements, which can be adjusted by the solid content and viscosity of PI solution, the type of coating spreader. Film thickness was measured by a thickness tester (C640, Labthink, Ji’nan, China) using a mechanical contact method with high precision.

## 3. Results and Discussion

### 3.1. Synthesis and Characterization of Semi-Alicyclic Polyimides

As shown in Scheme 1, five soluble semi-alicyclic PIs were prepared by solution polycondensation with equimolar alicyclic dianhydrides and aromatic diamines containing trifluoromethyl. PAA solutions were synthesized in the strong polar solvent of NMP at 30–40 °C, followed by the cyclodehydration reaction at elevated temperature. In view of relatively low chemical reactivity of alicyclic dianhydride, the polymerization from PAA to PI is generally recommended at high temperature to synthesize semi-alicyclic PIs with high molecular weight [38,39]. Correspondingly, PIs were gradually thermally imidized at 160–180 °C using base catalyst and azeotropic distillation with toluene to remove water produced as a byproduct during condensation reaction. The molecular weights and polydispersities of PIs were measured through GPC, and their results are summarized in Table 1.

The *M*_n_ and *M*_w_ of these PIs were in the range of 5.08–6.91 × 10^4^ g/mol and 7.54–9.23 × 10^4^ g/mol, respectively. All the semi-alicyclic PIs showed high molecular weight and narrow molecular weight distribution (*M*_w_/*M*_n_) below 1.50, which can reach the molecular weight level of fully-aromatic PIs and is better than most semi-alicyclic systems reported in the literatures [22,35]. This ensured their thermal and mechanical properties of corresponding films. In addition, it is also noted thatsemi-alicyclic PIs have excellent solubility, which can be dissolved in the strong and even weak polar solvents including NMP, *N,N*-dimethylformamide (DMF), *N,N*-dimethylacetamide (DMAc), tetrahydrofuran (THF) and dioxane. The improved solubility of these PIs is thought to be the presence of alicyclic structures and relatively high fluorine content derived from the trifluoromethyl groups in diamine moiety. In addition, semi-alicyclic PI-5 derived from CBDA and 6FBAS showed much better solubility than CBDA-based PI with TFMB reported in the literature [28]. This is mainly because the incorporation of ether linkages and sulfonyl groups in diamine moiety. Thus, these soluble semi-alicyclic PIs can be easily processed by common organic solvents, which is very convenient for a variety of applications.

**Figure 1:**
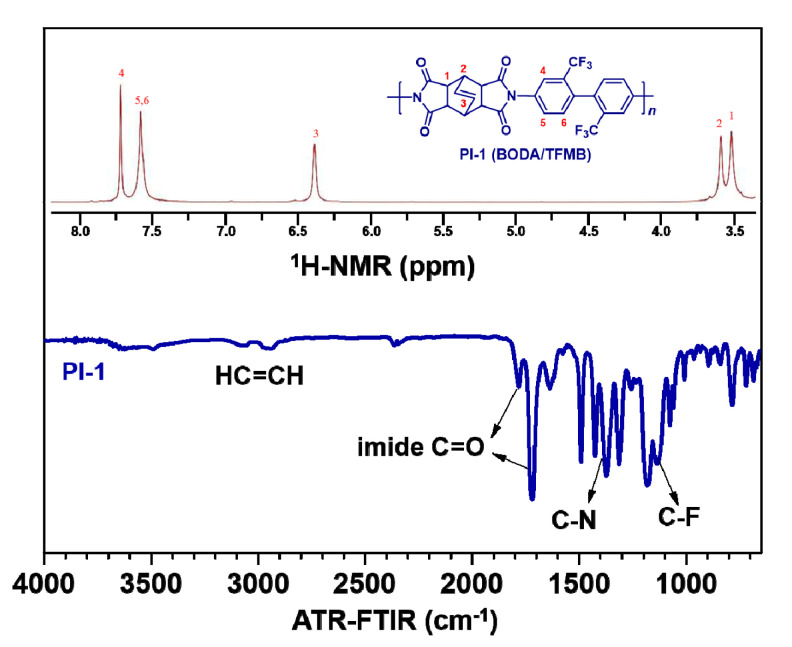
Attenuated total reflectance Fourier transform infrared spectroscopy (ATR-FTIR) and ^1^H-nuclear magnetic resonance (^1^H NMR) spectra of representative PI-1 film.

The imidization degree and chemical structures of semi-alicyclic PIs were characterized by Attenuated Total Reflectance Fourier Transform Infrared Spectroscopy (ATR-FTIR) and ^1^H-nuclear magnetic resonance (NMR) spectra, respectively. Figure 1 gives the ATR-FTIR and ^1^H NMR spectra of representative PI-1 film, and other ATR-FTIR spectra of semi-alicyclic PI films are shown in Figure S1 (shown in Supporting Information). The characteristic peaks of amide and carboxyl linkage around 3200–3400 cm^−1^ (N–H and COOH) and 1660 cm^−1^ (CO–NH) were not found, which qualitatively indicated that PAAs have been converted to PIs. Meanwhile, the absorption peaks assigned to the symmetric and asymmetric stretching vibration of carbonyl group (C═O) in imide ring, as well as imide C=N were observed at about 1780, 1715 and 1370 cm^−1^, respectively. The results also proved the successful imide conversion from amic acid groups. The degree of imidization can be quantitatively determined by the ratio of absorbance of imide C–N at 1370 cm^−1^ to that of C=C stretching at 1513 cm^−1^ [40]. All the values calculated by *I*_1370_/*I*_1513_ were more than 99% for these semi-alicyclic PIs. It is indicated that the precursor PAAs were successfully converted to corresponding PIs with a nearly complete imidization. Besides, the specific absorptions of C–F stretching vibration were detected around 1250 and 1135 cm^−1^ due to trifluoromethyl groups in the structure.

Chemical structures of semi-alicyclic PIs can be further identified by ^1^H NMR measurement. As depicted in Figure S2 (shown in Supporting Information), all the hydrogen protons can be clearly clarified in the ^1^H-NMR spectra. The characterization results demonstrate that semi-alicyclic PIs with expected chemical structures were successfully prepared through high temperature solution polycondensation. Besides, the surface and internal morphology of PI films were analyzed by scanning electron microscopy (SEM). SEM images of surface and cross-sectional fracture of representative PI-1 film are given in Figure S3 (shown in Supporting Information). It is observed that the film surface is smooth and flat, and there are no defects found inside such as bubbles or cracks.

Water absorption (*W*_A_) of PI films is an important factor for application in manufacturing flexible optoelectronic devices. As summarized in Table 1, *W*_A_ values of semi-alicyclic PI films were quantitatively evaluated. These PI films with fluorine content above 14.6% showed significantly low *W*_A_ values ranging from 0.77% to 1.08%, whereas the typical commercial PI film Kapton derived from pyromellitic dianhydride (PMDA) and 4,4’-diaminodiphenyl ether (4,4’-ODA) had a high *W*_A_ value around 2.65%. It is indicated that hydrophobic trifluoromethyl groups and nonpolar alicyclic moieties in the mainchain can effectively inhibit the water adsorption on the film surface, and prevent water molecules from further entering the internal structure of film. Moreover, water contact angle (CA) measurements of semi-alicyclic PIs were performed to reflect their hydrophobicity that is important for application as substrates. Water contact angle images are given in Figure S4 (shown in Supporting Information) with the data listed in Table 1. The intrinsic CA values of these PIs ranged from 89.2° to 93.7°, which were remarkably increased compared to Kapton film (79.3°). These CA values were positively correlated with fluorine content. It is indicated that hydrophobicity of semi-alicyclic PIs can be enhanced due to the incorporation of trifluoromethyl. This inference is consistent with the above water absorption results.

### 3.2. Mechanical Properties and Thermal Diffusivities of Semi-alicyclic Polyimides

The mechanical properties of PI films are associated with their flexibility and rigidity of molecular chains. Tensile strength (*T*_S_), modulus (*T*_M_) and elongation at the break (*E*_B_) for semi-alicyclic PIs were evaluated, and the results are listed in Table 2. All the PI films exhibited high *T*_S_ values in the range of 77.1–97.9 MPa, combined with moderate *T*_M_ values around 2.0–2.9 GPa and *E*_B_ values from 5.1% to 10.1%. It is noted that the TFMB-based PI-1 film showed the highest *T*_S_ and the lowest *E*_B_ values, indicating that its molecular chain is linear and rigid. In contrast, the 6FBAB-based PI-3 film had the lowest *T*_S_ and the highest *E*_B_ values due to the incorporation of a flexible ether bond. Besides, it is also found that the sulfone-containing PIs i.e., PI-4 and PI-5 films displayed enhanced mechanical properties. The result means that introducing polar groups into the mainchain can improve intermolecular interactions and thus have a great influence on the mechanical performance of PIs. As comparing with PIs based on different dianhydrides including BODA and CBDA, the films showed major differences on their strength and modulus performance. The BODA-based PI-4 film exhibited higher *T*_S_ and lower *T*_M_ values than CBDA-based PI-5 film. This could be ascribed to the combination effect of rigidity and linearity of molecular chains. The dianhydride CBDA having relatively linear structure endowed PI chain with more rigidity, thus obtaining higher modulus. As depicted above, these semi-alicyclic PI films have adjustable and sufficient toughness for application as flexible substrates.

The out-of-plane thermal diffusivities (*D*_⊥_) of semi-alicyclic PI films are also given in Table 2. It is reported that the intrinsic thermal conductivity of PIs depends on their molecular structure, chain orientation and molecular packing [41,42]. Compared with the *D*_⊥_ value (11.5 × 10^−8^ m^2^/s) of fully-aromatic Kapton film, semi-alicyclic PI films showed much lower *D*_⊥_ values below 6.0 × 10^−8^ m^2^/s. Because of the directional dependence of average phonon velocity in PI films, thermal energy is transferred more efficiently through covalent bonds and aromatic rings along the molecular chains, compared to the weakly van der Waals interactions. Moreover, fully-aromatic PIs with linear and rigid mainchains undoubtedly have higher degrees of molecular orientation along the out-of-plane direction, which contribute to enhance the *D*_⊥_ values of films. Among these semi-alicyclic PI films, it is further found that PI-1 and PI-3 exhibited relatively low *D*_⊥_ values due to the rigid biphenyl units at the diamine moieties, while the PI-2 and PI-4 with bent and rotatable linkages (ether or sulfonyl groups) showed large values even having same dianhydride. Besides, the CBDA-based PI-5 exhibited lower *D*_⊥_ value than BODA-based PI-4, although they had the same diamine. Just as explained previously, CBDA moiety has relatively more linear and rigid molecular structure, which results in a preferred chain orientation along the in-plane direction rather than out-of-plane direction.

### 3.3. Thermal Properties of Semi-Alicyclic Polyimides

The dynamic mechanical analysis (DMA), thermogravimetric analysis (TGA) and thermomechanical analysis (TMA) were used to investigate the effect of alicyclic structures and trifluoromethyl groups on thermal properties of semi-alicyclic PIs. All the thermal results of semi-alicyclic PIs are summarized in Table 3. As shown in Figure S5 (shown in Supporting Information), the heat resistance of PIs is reflected by *T*_g_ values recorded as the peak temperature of *tan δ* curves in DMA. These PI films exhibited *T*_g_ values in the range of 285–390 °C, especially the TFMB-based PI-1 showing the highest heat resistance. It was found that the *T*_g_ values decreased in the order of diamine moieties: TFMB > 6FBAB > 6FBAS > 6FAPB. This phenomenon can be ascribed to the rigidity degree of backbones. The rigid biphenyl unit is beneficial to the improved heat resistance, especially in the absence of any other bent or rotatable linkages. PI-2 derived from 6FAPB has the highest amount of flexible ether bonds in diamine moiety, consequently exhibiting the lowest *T*_g_ value. Besides, different alicyclic dianhydride moieties had almost no influence on the glass transition. PI-4 and PI-5 based on different dianhydrides showed their *T*_g_ values around 295 °C. Compared with those similar CBDA-based PIs with 6FAPB or 6FBAB reported in the literature [29], PI-5 displayed significantly improved heat resistance with *T*_g_ increased by nearly 50 °C. This can be ascribed to the introduction of sulfonyl groups and reduction of flexible ether bonds in backbones.

**Figure 2:**
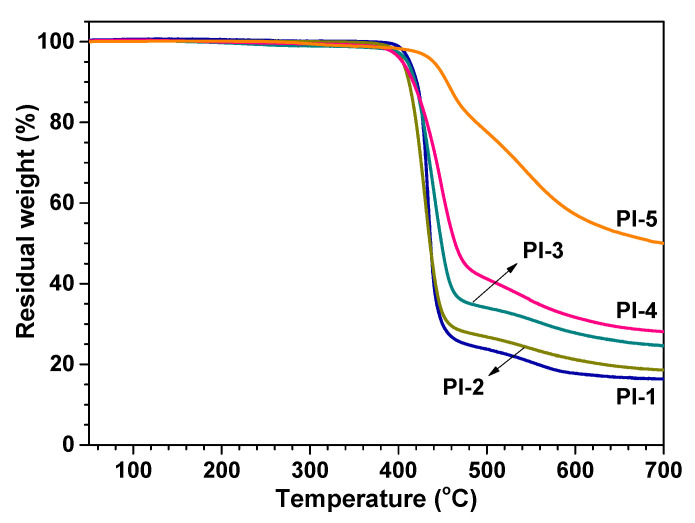
Thermogravimetric analysis (TGA) curves of semi-alicyclic polyimide films in nitrogen.

TGA curves of semi-alicyclic PIs in nitrogen are illustrated in Figure 2, and the onset, 5 wt% and 10 wt% weight loss decomposition temperatures (*T*_onset_, *T*_5%_ and *T*_10%_) are taken to evaluate their thermal stabilities. For these semi-alicyclic PIs, there is no obvious weight loss below 350 °C, suggesting PI films have been completely imidized and there is almost no solvent residue. This is in accordance with the above analysis of infrared spectroscopy. Compared with fully-aromatic PIs with decomposition temperatures over 500 °C, semi-alicyclic PI films exhibited moderate thermal endurance property with *T*_onset_ and *T*_5%_ values in the range of 411–436 °C and 405–440 °C, respectively. PI-5 film displays the highest *T*_10%_ value of 455 °C. In the previous work, it has been reported that semi-alicyclic PIs based on alicyclic dianhydrides have inferior thermal stability compared to fully-aromatic PIs [37]. In contrast with 6FAPB-based PI-2, fully-aromatic PI derived from pyromellitic dianhydride (PMDA) and 6FAPB exhibited *T*_5%_ value around 541 °C, which is 136 °C higher than semi-alicyclic former [43]. It is revealed that thermal stabilities of semi-alicyclic PIs are greatly influenced by the alicyclic structures of dianhydrides, which can significantly reduce the decomposition temperature. For the PIs derived from dianhydride BODA with different aromatic diamines, there are no major changes between their *T*_onset_ values from 411 °C to 422 °C. Among them, PI-1 and PI-2 respectively exhibited the highest and the lowest values due to their rigid or flexible units in diamine moieties. Moreover, it is found that CBDA-based PI-5 showed *T*_onset_ value up to 436 °C and residual weight retention at 700 °C (*R*_700_) around 50%, representing apparently better thermal stability with 14–25 °C and 22–34% higher than thoseof BODA series. This is mainly related to the rigidity and linearity of dianhydride moiety.

Thermal dimension stability is another key factor for PI films when applied as flexible substrates. As shown in Figure S6 (shown in Supporting Information), thermal expansion behavior of semi-alicyclic PIs was investigated, and in-plane CTE values in the temperature range of 50–200 °C are listed in Table 3. The PI films exhibited moderate in-plane CTE values in the range of 55–64 ppm/°C, except for PI-1 derived from TFMB. It is notable that the CTE values of PI films are greatly affected by the rigidity or linearity of molecular chain [44,45]. PI-1 film showed excellent dimension stability with CTE below 35 ppm/°C due to its rigid and linear biphenyl bond in the repeating unit. In summary, it is demonstrated that there is an evident relationship between thermal properties of PIs and the rigidity or linearity of structures. Although the glass transition, decomposition and dimension stabilities of PI films are all reduced to some extent when introducing flexible or bent linkages, the overall thermal properties of these semi-alicyclic PIs remain at a moderate level, which are sufficient for most applications as flexible substrates.

### 3.4. Refractive and Dielectric Properties of Semi-Alicyclic Polyimides

The refractive performance of semi-alicyclic PIs was studied by prism coupling method. The in-plane and out-of-plane refractive indices (*n*_∥_ and *n*_⊥_) were measured at 632.8 nm, and the average refractive indices (*n*_av_) and birefringence (∆*n*) were also calculated. All the refractive results are summarized in Table 4. The *n_av_* values of semi-alicyclic PIs ranged from 1.5418 to 1.5833, obviously lower than that of typically fully-aromatic Kapton film (1.7108). It can be explained by the polarizability of component atoms in polymer backbones. In general, higher polarizability provides a higher refractive index because of the greater dipole moment under electromagnetic field. The fluorine atom shows high electronegativity and small volume, thus resulting in a relatively low polarizability, while the carbon atom presents large polarizability [46]. Therefore, the introduction of low polarizable atoms or chemical bonds such as alicyclic structures and trifluoromethyl groups can significantly reduce their refractive indices. Especially PI-1 film derived from TFMB diamine gave the lowest *n*_∥_, *n*_⊥_ and *n_av_* value, which can be attributed to both the highest content of low polarizable fluorine atoms and more loose chain arrangement due to bulky side groups.

Optical birefringence (∆*n*) of polymer film is defined as the difference between the in-plane and out-of-plane refractive indices. It is a significant parameter to reflect the anisotropy of refractive index and generally used to estimate the orientation degree of polymer chains in the film plane [47,48]. As listed in Table 4, these semi-alicyclic PI films exhibited low ∆*n* values in the range of 0.0155–0.0247. In contrast, fully-aromatic Kapton film showed high ∆*n* value of 0.0485. It is also found that there are some differences for PIs with different diamine or dianhydride moieties. For instance, among the PI films based on BODA dianhydride, PI-2 showed the lowest ∆*n* values with isotropic and random chain orientation. This can be ascribed to the bent and flexible ether linkage in diamine moiety, resulting that PI film tends to have a smaller fraction of oriented chains along the in-plane direction. Furthermore, for PI films based on 6FBAS diamine, the CBDA-based PI-5 exhibited a remarkable ∆*n* value exceeding 0.024, higher than that of BODA-based PI-4. It can be assumed that the rigid-rod structure of CBDA moiety causes the enhanced chain orientation in the film plane. The optical anisotropy of films is an important factor for various optoelectronic applications such as flexible display. These semi-alicyclic PI films with low optical anisotropy can prevent color distortion and leakage of light depending on the viewing angle.

Based on the measured refractive indices, the optical dielectric constants (*ε*_opt_) and the anisotropy values (∆*ε*) of semi-alicyclic PIs were estimated by Maxwell equation and summarized in Table 4. The *ε*_opt_ values obtained at the optical frequency were in the range of 2.61–2.76, which showed a dependence on the backbones of PIs. With the increasing fluorine content, the *ε*_opt_ values of these films are gradually reduced. TFMB-based PI-1 with the highest fluorine content of 21.4% exhibited the lowest *ε*_opt_ value of about 2.61, while 6FBAS-based PI-4 with fluorine content of 14.6% possessed an increased *ε*_opt_ value around 2.73. Furthermore, fully-aromatic Kapton film without any fluorine atom had a relatively high *ε*_opt_ value up to 3.22. It is noteworthy that the dielectric property is mainly attributed to electronic polarization and partly to atomic polarization. The dielectric constants of PIs can be reduced both by incorporating low polarized atoms and further by increasing molecular free volume via the bulky side groups. For these semi-alicyclic PIs, their dielectric constants were significantly reduced by the combined effect of low polarizabilities and large bulkiness due to the presence of nonpolar alicyclic units and trifluoromethyl groups. In addition, the ∆*ε* values are used to represent the in-plane and out-of-plane dielectric constant differences of PI films. For the microelectronic devices, the large anisotropy in the dielectric constant can cause near-coupled-noise problem due to the unwanted crosstalk. Compared with Kapton film, these semi-alicyclic PI films showed obviously low ∆*ε* values. PI-3 with the most flexible linkages in the mainchain exhibited the lowest dielectric anisotropy. Moreover, it is found that there is no linear relationship between ∆*ε* and fluorine content, which is different from the phenomenon of dielectric constant. With the low dielectric data and related anisotropy, semi-alicyclic PIs can be good candidate materials for microelectronic devices.

### 3.5. Optical Properties of Semi-Alicyclic Polyimides

**Figure 3:**
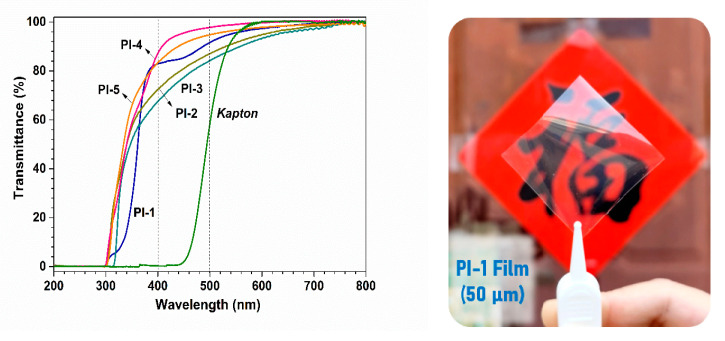
UV–Vis spectra of semi-alicyclic polyimide films and the appearance of PI-1 film.

The optical transparency of PI films is crucial in many optoelectronic applications that require transparency, such as optical waveguide, flexible displays and transparent circuit. The optical properties of semi-alicyclic PI films were respectively evaluated by UV–Vis spectra and color intensities. Figure 3 shows the UV–Vis spectra of these PI films at 25 ± 3 μm thickness, and the transmittance dataat different wavelength are summarized in Table 5. It is noted that semi-alicyclic PI films were highly transparent with cutoff wavelength (*λ*_0_) in the range of 298–313 nm and transmittance at 500 nm (*T_5_*_00_) above 85%. In contrast, fully-aromatic Kapton film with the same thickness showed a remarkably high *λ*_0_ value up to 444 nm that dramatically lowered its optical transmittance below 500 nm. Compared with 2% transmittance at 450 nm (*T_45_*_0_) of Kapton film, these semi-alicyclic PI films exhibited extremely optical improvement. In particular, PI films derived from TFMB or 6FBAS series showed better optical transparency in the visible region no matter BODA or CBDA dianhydride.

The difference of optical transparency among PI films was further evaluated by their color intensities calculated according to the CIE LAB equation. Semi-alicyclic PI films with a unified thickness of 50±3 μm were measured and the results are listed in Table 5. The parameters of color intensities include the lightness (L*), redness (a*), and yellowness (b*) indices. The L* index varying from 0 to 100 refers to lightness change from black to white. The positive or negative a* index indicates red or green color. Likewise, the positive or negative b* index reflects yellow or blue color. All the semi-alicyclic PI films exhibited higher lightness with L* values over 89.4 than Kapton film with L* of 79.6. Meanwhile, these PI films showed a* values of nearly zero combined with much lower b* values in the range of 3.4–4.2. It is indicated that semi-alicyclic PI films displayed not only excellent transparence but also essentially colorless appearance, which can be seen from the appearance of representative PI-1 film shown in Figure 3. In contrast, the Kapton film with deep yellow to brown coloration exhibited a high a* value of 6.3 and a very large b* value over 107. It is known that optical transparency and coloration of PI films are mainly dependent on their molecular structures. The improved optical transparency of these films can be ascribed to the incorporation of alicyclic moieties and electron-withdrawing side groups. The combined effects are beneficial to suppress or eliminate CTC between inter- and intra-molecular chains that result in the characteristic coloration of fully-aromatic PIs.

**Figure 4:**
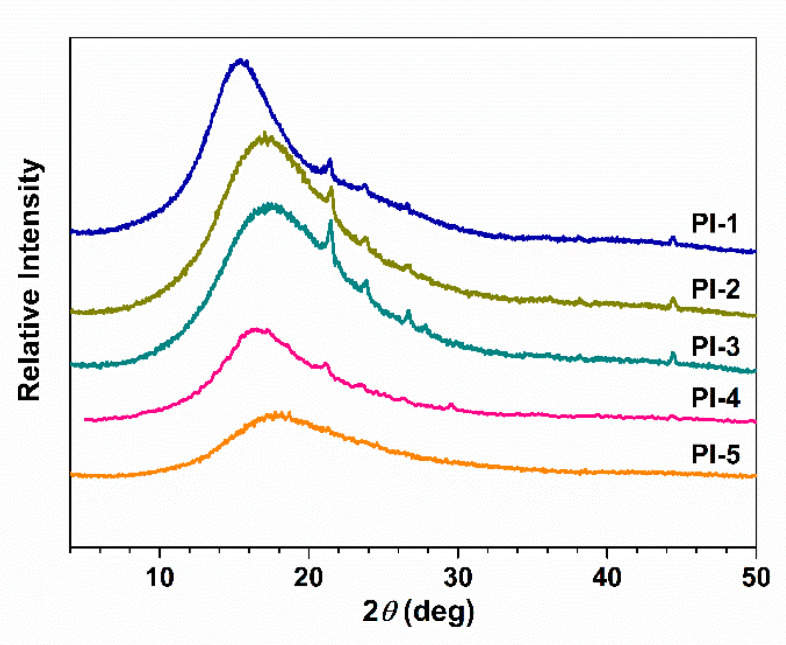
Wide angle X–ray diffractometry (WAXD) profiles of semi-alicyclic polyimide films.

To further clarify the effect of backbone structures and side groups on the optical transparency, the aggregation structures of semi-alicyclic PI chains was investigated by WAXD based on the reflection mode. WAXD patterns of PIs are illustrated in Figure 4, and the mean interchain distance (*d*) values are calculated from the maxima of diffraction peaks by Bragg’s equation. Besides, molecular orbital energies of model compounds for the repeating units of PIs were also determined by density functional theory (DFT) calculations using a large basis-set function [49,50]. The model compound structures of semi-alicyclic PIs are listed in Figure S7 (shown in Supporting Information).

It is observed that all the semi-alicyclic PI films exhibited broad and featureless peaks like the amorphous patterns with large full width at half peak maximum. This phenomenon reveals that the linearity and rigidity of PI chains could be disturbed by the steric hindrance of bulky substitutes or rotational ether bonds with twisted conformation. The interchain distance *d* values are used to quantitatively present the packing of molecular chains. It is noticeable that PI-1 derived from TFMB diamine showed the largest *d* value up to 5.7 Å, whereas PI-3 and PI-5 exhibited small *d* values around 5.0 Å similar with that of Kapton film. Among PIs based on the same BODA dianhydride, the *d* values were in the decreasing order of TFMB>6FBAS>6FAPB>6FBAB. This order is basically consistent with the optical transmittance of these PI films, suggesting that optical properties are affected by the packing or aggregation of molecular chains to some extent. It is demonstrated that bulky trifluoromethyl substituents effectively hinder the dense packing of chains and remarkably expand intermolecular distances of PIs. This is obviously beneficial for the elimination of CTC to improve transparency and reduce color of films.

According to the calculated molecular orbital energies of model compounds, the electron accepting abilities of dianhydride moieties and donating abilities of diamine moieties in various PIs can be obtained. These molecular orbital parameters including the lowest unoccupied molecular orbital energy (*E*_LUMO_), the highest occupied molecular orbital energy (*E*_HOMO_) and energy band gap (*E*_GAP_) are summarized in Table 6. Compared with fully-aromatic Kapton, all the semi-alicyclic PIs exhibited much higher *E*_LUMO_ values (from −1.89 eV to −1.42 eV), while having lower *E*_HOMO_ values below −6.41 eV. It is indicated that the electrophilicity of alicyclic dianhydride moieties is obviously reduced, and the donating ability of trifluoromethyl-containing aromatic diamine moieties is also not high. Consequently, these semi-alicyclic PIs showed significantly large energy differences between *E*_LUMO_ and *E*_HOMO_ compared to fully-aromatic PIs. Among these PIs, PI-1 and PI-3, respectively, exhibited the largest and the lowest *E*_GAP_ values of 5.43 eV and 4.92 eV. In contrast, the Kapton displayed much lower *E*_GAP_ value of 2.88 eV. It is known that a large *E*_GAP_ value generally means a high electron excitation energy. It is favorable to destroy the CTC formation and lead to weak absorption edge accompanied with high optical transmittance. The results of molecular orbital energies are in accordance with the optical transparency of semi-alicyclic PI films discussed above, suggesting that both inter- and intra-molecular charge transfer interactions are effectively suppressed. That is the main reason why the incorporation of alicyclic structures and trifluoromethyl groups can improve optical transmittance and eliminate coloration of films.

### 3.6. Structure–Property Relationships of Semi-Alicyclic Polyimides

**Figure 5:**
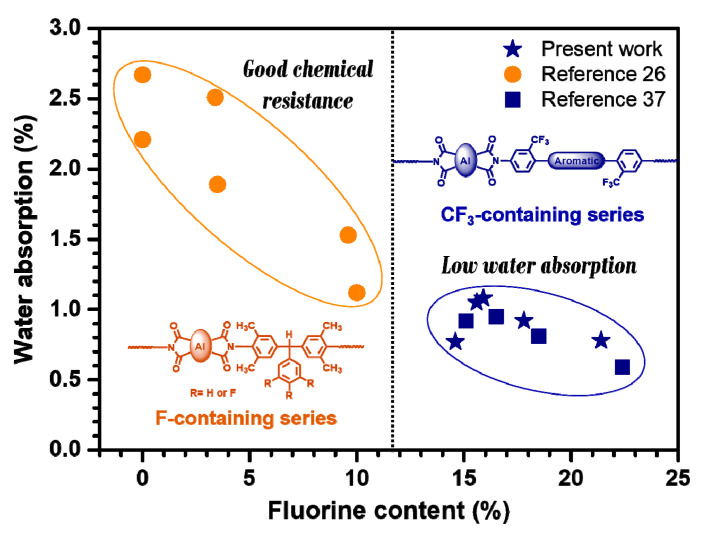
The correlation between fluorine content and water absorption of semi-alicyclic polyimides and comparison with other semi-alicyclic systems reported in the previous work [26,37].

Based on the systematic investigation of performance, we try to provide the structure–property relationships for fluorinated semi-alicyclic PIs. The different effects of trifluoromethyl and fluorine atoms on performance of semi-alicyclic PIs were also compared with results reported previously [26,37]. The representative structures and performance data of semi-alicyclic PIs for comparison are summarized in Table S1 (shown in Supporting Information), including the systems prepared in the present work and those systems with the same alicyclic BODA or the same fluorinated diamines reported in the previous work. The correlation between fluorine content and water absorption for these different PIs was depicted in Figure 5. As expected, semi-alicyclic PIs containing trifluoromethyl groups exhibit much lower water absorption than those containing fluorine atoms, which is ascribed to the level of fluorine content. Trifluoromethyl groups obviously contribute to higher fluorine content that is very beneficial to reduce water absorption of films due to strong hydrophobicity. On the other hand, although the low fluorine content is detrimental for lowering water absorption, it is effective to improve chemical resistance of films. Those semi-alicyclic PIs with low fluorine content below 10% show good chemical resistance to commonly used solvents in the process of devices manufacturing, such as methyl ethyl ketone (MEK) and potassium hydroxide solution (10wt%). However, those semi-alicyclic PIs containing trifluoromethyl groups display bad chemical resistance to MEK even though it is no problem for potassium hydroxide solution. It is indicated that excessive fluorine content will sacrifice chemical resistance of films. Thus, controlling fluorine content at an appropriate level is the key to achieving balance between water absorption and chemical resistance for semi-alicyclic PIs. Considering that the optical transparency of films must be guaranteed, it may be an effective method to adjust fluorine content of semi-alicyclic PIs containing trifluoromethyl by copolymerization with other fluorine-free diamines.

**Figure 6:**
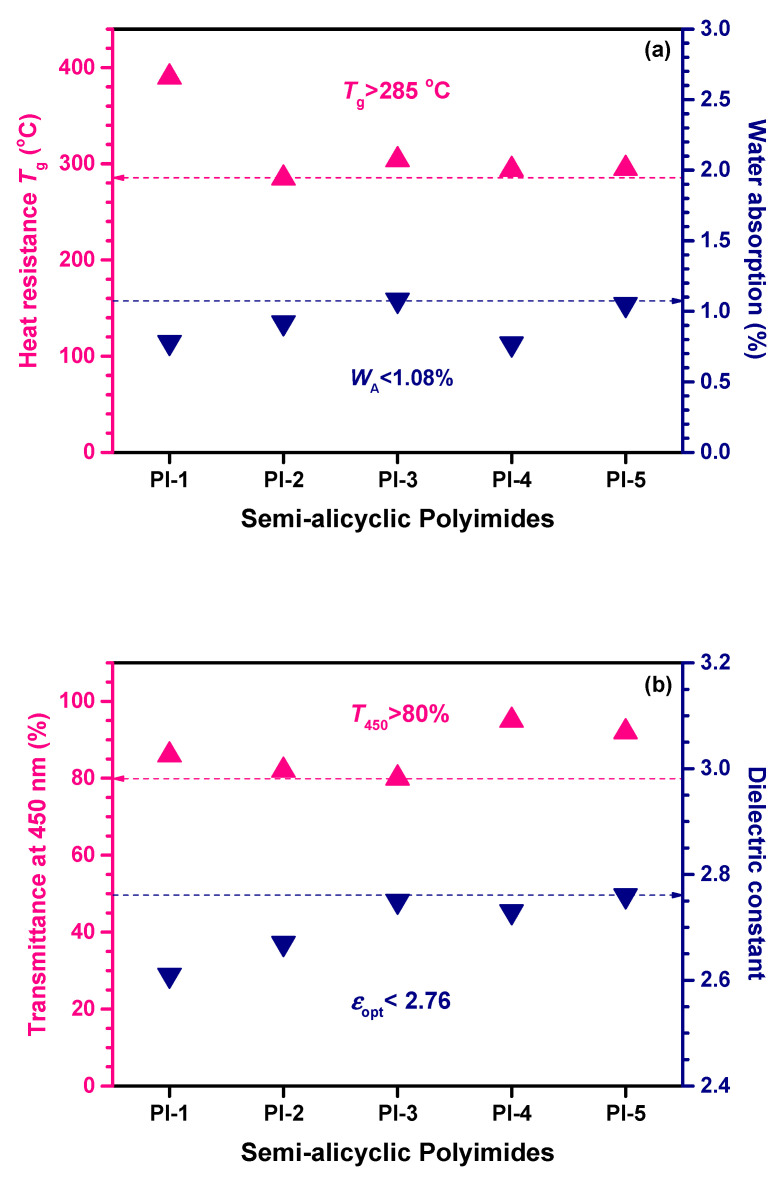
The performance of semi-alicyclic polyimides prepared in the present work.

As shown in Figure 6a, heat resistance (represented as *T*_g_) of these PIs was not sacrificed in the presence of alicyclic structures. In contrast, structures of diamines have a major impact on their heat resistance. PIs with more linear and rigid backbones will have higher *T*_g_ values. It is proved that flexible ether bond with highly distorted molecular conformation significantly affects the heat resistance of PIs. Diamines containing ether linkages such as 6FAPB and 6FBAB result in lower *T*_g_ values for semi-alicyclic PIs, regardless of the structures of cycloaliphatic dianhydrides. Meantime, similar structure–performance laws also apply to thermal dimension stability (represented as CTE) of semi-alicyclic PIs. Therefore, as long as linear and rigid structure is used in the design of diamine moiety, highly heat-resistant semi-alicyclic PIs with outstanding thermal dimension stability can be obtained. Particularly, BODA-based and TFMB-based PIs showed higher *T*_g_ and lower CTE values.

The intense electron-withdrawing characteristics of trifluoromethyl groups are also outstanding in improving optical transparency and decreasing dielectric properties, which is illustrated in Figure 6b. All the semi-alicyclic PIs exhibit high light transmittance and obviously low dielectric constants. They are mainly ascribed to the synergistic effects of alicyclic structures and fluorinated groups. It is further observed that structures of diamines have an effect on transparency or dielectric properties of PIs. For instance, sulfone-containing diamine 6FBAS can contribute positively to the improved optical transparency except for maintaining low dielectric constant. Besides, compared with fluorinated semi-alicyclic PIs derived from BODA or H-PMDA series prepared in the present work and other references [26,37], it can be found that diamine containing trifluoromethyl groups rather than fluorine atoms or cycloaliphatic H-PMDA is more beneficial to obtain highly transparent films. The above results also indicate that, for those fluorinated semi-alicyclic PIs, other desired performance can be achieved through further structure design on the basis of intrinsic high transparency and low dielectric properties.

In addition, in the reported literature, the toughness results of semi-alicyclic PIs have not always been examined because cycloaliphatic structures often yield brittle films. It is not easy to overcome the embrittlement problem for alicyclic-based PIs. From the mechanical performance shown in Table S1, it can be seen that roughness of semi-alicyclic PIs can be effectively enhanced by introducing flexible ether bonds in the diamine moiety. Compared with rigid PI-1 system, the elongation values of those flexible PIs were nearly doubled. Thus, it is a facile way to adjust diamine structures to improve roughness of semi-alicyclic PI films without sacrificing their polymerization activity or process conditions.

## 4. Conclusions

Based on alicyclic dianhydrides and trifluoromethyl-containing aromatic diamines, a series of semi-alicyclic PIs with fluorine content above 14.6% was successfully prepared. All the PI films exhibited remarkably low water absorption below 1.08% and good mechanical properties with tensile strength up to 97.9 MPa. These PI films also showed high heat-resistance with *T_g_* from 294 °C to 390 °C, having moderate thermal stability with onset decomposition temperatures above 411 °C. Especially, PI-1 derived from TFMB diamine simultaneously exhibited high *T_g_* of 390 °C and low in-plane CTE around 34 ppm/°C due to its linear and rigid backbone. It is further found that these PIs showed obviously low refractive indices and optical birefringence. The dielectric constants were significantly reduced to 2.61–2.76 because of the low polarizability of alicyclic units and large volume effect of trifluoromethyl groups. Besides, the semi-alicyclic PI films were highly transparent with cutoff wavelength in the range of 298–313 nm and transmittance at 500 nm above 85%. These films possessed essentially colorless appearance with b* values below 4.2. The remarkable optical improvement is ascribed to the weak electron-accepting alicyclic moieties and bulky electron-withdrawing trifluoromethyl or sulfone groups. The present work provides an effective strategy to design molecular structures of optically transparent PIs with desired properties, including the outstanding solubility, high heat resistance, low refractive index and dielectric constant with almost negligible anisotropy.

## Figures and Tables

**Table 1:** Physical properties of semi-alicyclic polyimides ^a^.

PIs	Molecular Weight			Fluorine Content (%)	*W*_A_ (%)	CA (°)
	*M*_n_ × 10^4^(g/mol)	*M*_w_ × 10^4^ (g/mol)	*M*_w_/*M*_n_			
PI-1	5.84	8.35	1.43	21.4	0.78	93.7
PI-2	5.86	8.32	1.42	17.8	0.92	92.8
PI-3	5.08	7.54	1.48	15.9	1.08	89.7
PI-4	6.77	9.23	1.36	14.6	0.77	89.2
PI-5	6.91	8.76	1.27	15.6	1.05	89.4

^a^*M*_n_ and *M*_w_: the number and weight average molecular weight, measured in *N,N*-dimethylformamide (DMF) by gel permeation chromatography (GPC) with multiangle laser light scattering system; *W*_A_: water absorption rate at 25 °C for 24 h; CA: water contact angle.

**Table 2:** Mechanical properties and thermal diffusivities of semi-alicyclic polyimide films ^a^.

PIs	*T*_S_ (MPa)	*T*_M_ (GPa)	*E*_B_ (%)	*D*_⊥_ (×10^−8^ m^2^/s)
PI-1	97.9 ± 2.1	2.7 ± 0.1	5.1 ± 0.4	4.7
PI-2	84.4 ± 3.5	2.1 ± 0.2	8.0 ± 0.4	6.0
PI-3	77.1 ± 2.8	2.0 ± 0.1	10.1 ± 0.4	5.0
PI-4	94.5 ± 2.0	2.3 ± 0.1	9.1 ± 0.6	5.9
PI-5	90.6 ± 3.0	2.9 ± 0.1	8.0 ± 0.5	5.0

^a^*T*_S_: tensile strength, *T*_M_: modulus, *E*_B_: elongation at break; *D*_⊥_: out-of-plane thermal diffusivities.

**Table 3:** Thermal properties of semi-alicyclic polyimide films ^a^.

PIs	*T*_g_ (°C)	*T*_onset_ (°C)	*T*_5%_ (°C)	*T*_10%_ (°C)	*R*_700_ (%)	CTE (ppm/°C)
PI-1	390	422	412	421	16.4	34
PI-2	285	411	405	412	18.5	62
PI-3	304	416	410	419	24.6	55
PI-4	294	418	405	417	28.1	63
PI-5	295	436	440	455	50.0	64

^a^*T*_g_: the glass-transition temperature; *T*_onset_, *T*_5%_ and *T*_10%_: the onset, 5% and 10% weight loss decomposition temperatures; *R*_700_: residual weight retention at 700 °C in nitrogen; CTE: in-plane coefficient of thermal expansion in the 50–200 °C range.

**Table 4:** Refractive indices and dielectric constant of semi-alicyclic polyimide films ^a^.

PIs	*n*_∥_	*n*_⊥_	*n_av_*	∆*n*	*ε*_opt_	∆*ε*
PI-1	1.5491	1.5273	1.5418	0.0218	2.61	0.0738
PI-2	1.5641	1.5486	1.5589	0.0155	2.67	0.0531
PI-3	1.5909	1.5615	1.5811	0.0294	2.75	0.1019
PI-4	1.5829	1.5640	1.5766	0.0189	2.73	0.0654
PI-5	1.5916	1.5669	1.5833	0.0247	2.76	0.0858
*Kapton*	1.7270	1.6785	1.7108	0.0485	3.22	0.1817

^a^*n*_∥_, *n*_⊥_ and *n_av_*: the in-plane, out-of-plane and average refractive indices respectively, *n_av_* = (2*n*_∥_ + *n*_⊥_)/3; Δ*n*: birefringence, ∆*n* = *n*_∥_ − *n*_⊥_; *ε*_opt_: optical dielectric constant estimated by average refractive index method, *ε*_opt_ = 1.1*n_av_*^2^; Δ*ε*: the anisotropy between in-plane and out-of-plane dielectric constant, Δ*ε* = *ε*_∥_ − *ε*_⊥_= 1.1(*n*_∥_^2^ − *n*_⊥_^2^).

**Table 5:** Optical properties of semi-alicyclic polyimide films ^a^.

PIs	Transmittance ^a^				Color Parameters ^b^		
	*λ*_0_ (nm)	*T*_400_ (%)	*T*_450_ (%)	*T_5_*_00_ (%)	L*	a*	b*
PI-1	298	83	86	93	89.4	−0.2	3.8
PI-2	303	74	82	88	91.0	−0.1	4.0
PI-3	313	68	80	85	91.7	−0.3	4.2
PI-4	299	87	95	98	92.3	−0.2	3.4
PI-5	301	84	92	95	91.2	−0.1	4.0
*Kapton*	444	0	2	58	79.6	6.3	107.7

^a^ Film thickness: about 25 ± 3 μm; *λ*_0_: UV cutoff wavelength; *T*_400_, *T*_450_ and *T*_500_: transmittance at 400, 450 and 500 nm, respectively. ^b^ Film thickness: about 50 ± 3 μm; color parameters L*, a* and b*: calculated according to the CIE LAB equation.

**Table 6:** WAXD data and molecular orbital energies of semi-alicyclic polyimides.

Model Compounds	2*θ* (deg)	*d* (Å)	*E*_LUMO_ (eV)	*E*_HOMO_ (eV)	*E*_GAP_ (eV)
BODA/TFMB (PI-1)	15.3	5.7	−1.55	−6.98	5.43
BODA/6FAPB (PI-2)	16.9	5.2	−1.42	−6.63	5.21
BODA/6FBAB (PI-3)	17.4	5.0	−1.49	−6.41	4.92
BODA/6FBAS (PI-4)	16.5	5.4	−1.77	−6.83	5.06
CBDA/6FBAS (PI-5)	17.8	5.0	−1.89	−6.87	4.98
PMDA/ODA (Kapton)	18.1	4.9	−3.42	−6.30	2.88

*d*: interchain distances, calculated by Bragg’s equation, *d* = *λ*/(2sin*θ*); *E*_LUMO_: the lowest unoccupied molecular orbital energy, *E*_HOMO_: the highest occupied molecular orbital energy, *E*_GAP_: energy band gap, *E*_GAP_ = *E*_LUMO_ – *E*_HOMO_.

## Supplementary Materials

The following are available online at https://www.mdpi.com/2073-4360/12/7/1532/s1, Figure S1: ATR-FTIR spectra of semi-alicyclic polyimide films., Figure S2: ^1^H NMR spectra of the semi-alicyclic polyimides in in DMSO-*d**_6_*, Figure S3: Scanning electron microscopy (SEM) images of the surface and cross-sectional fracture of PI-1 film, Figure S4: Water contact angle images of the semi-alicyclic polyimide films; Figure S5: DMA curves of semi-alicyclic polyimide films in nitrogen, Figure S6: TMA curves of semi-alicyclic polyimide films in nitrogen, Figure S7: Model compound structures of semi-alicyclic polyimides for the DFT calculations, Table S1: Representative structures and performance comparison of different semi-alicyclic polyimides prepared in the present and previous work.
