# Supplementary material for: Preparation and Characterization of Semi-Alicyclic Polyimides Containing Trifluoromethyl Groups for Optoelectronic Application
Source: Polymers (Basel). 2020 Jul 11;12(7):1532. doi: 10.3390/polym12071532 (PMC7407393; doi:10.3390/polym12071532)
Supplement: Supplementary file 1 [file polymers-12-01532-s001.pdf]

Supporting Information

Preparation and Characterization of Semi-alicyclic Polyimides Containing Trifluoromethyl Groups for Optoelectronic Application

Mei Zhang [1,2,*], Wei-li Liu [1,2], Xia Gao [1,2], Peng Cui [1,2], Tao Zou [1,2], Guang-hui Hu [1,2], Li-ming Tao [3,*] and Lei Zhai [4]

[1] Beijing Center for Physical and Chemical Analysis, Beijing 100094, PR China
[2] Beijing Key Laboratory of Detection Technology and Quality Evaluation of Organic Material, Beijing 100094, PR China
[3] State Key Laboratory of Solid Lubrication, Lanzhou Institute of Chemical Physics, Chinese Academy of Sciences, Lanzhou, Gansu 730000, PR China
[4] Laboratory of Advanced Polymer Materials, Institute of Chemistry, Chinese Academy of Sciences, Beijing 100190, PR China
* Correspondence: zhangmei@bcpca.ac.cn (M. Zhang); taolm@licp.cas.cn (L.M. Tao).

Figure S1. ATR-FTIR spectra of semi-alicyclic polyimide films.

Figure S2. ^{1}H NMR spectra of the semi-alicyclic polyimides in in DMSO-d_6.

Figure S3. Scanning electron microscopy (SEM) images of the surface and cross-sectional fracture of PI-1 film.

Figure S4. Water contact angle images of the semi-alicyclic polyimide films.

Figure S5. DMA curves of semi-alicyclic polyimide films in nitrogen.

Figure S6. TMA curves of semi-alicyclic polyimide films in nitrogen.

Figure S7. Model compound structures of semi-alicyclic polyimides for the DFT calculations.

Table S1. Representative structures and performance comparison of different semi-alicyclic polyimides prepared in the present and previous work.

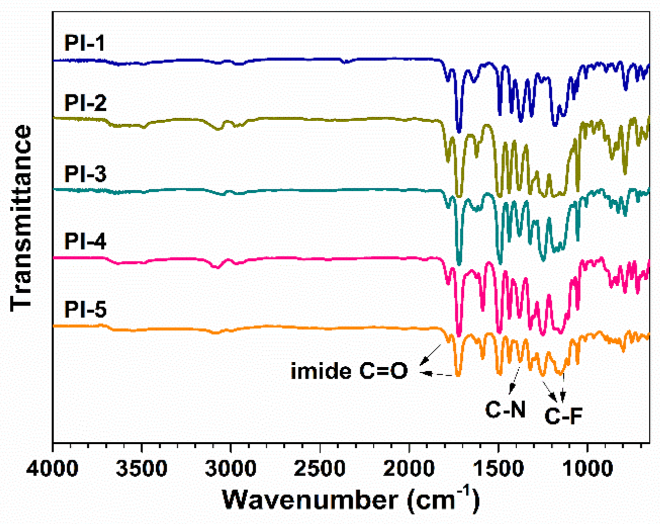

Figure S1. ATR-FTIR spectra of semi-alicyclic polyimide films.

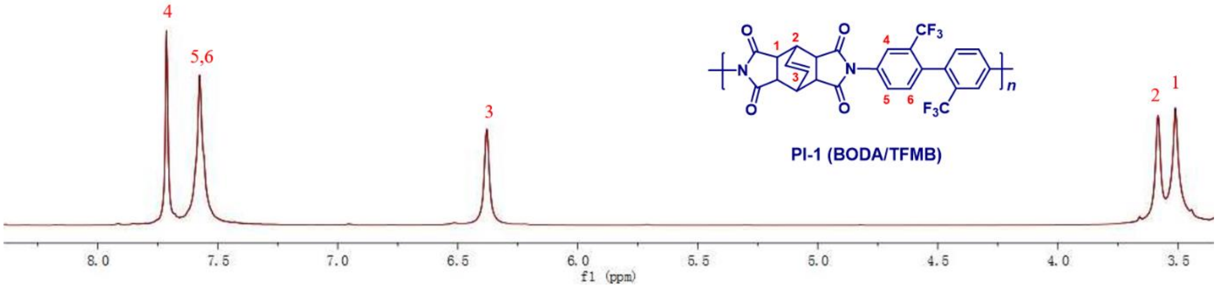

PI-1 (BODA/TFMB)
f1 (ppm)

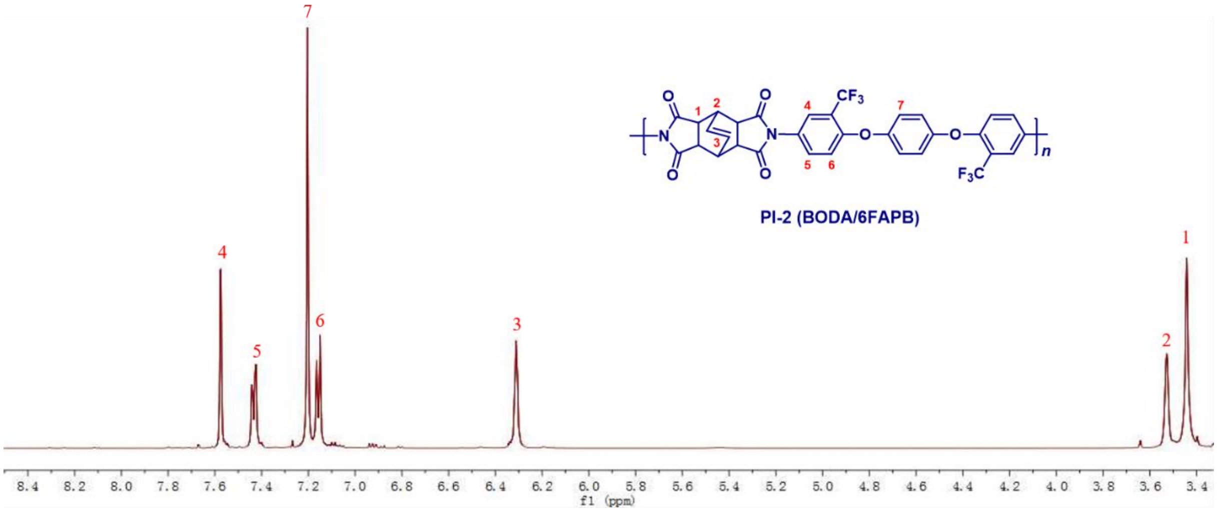

PI-2 (BODA/6FAPB)
f1 (ppm)

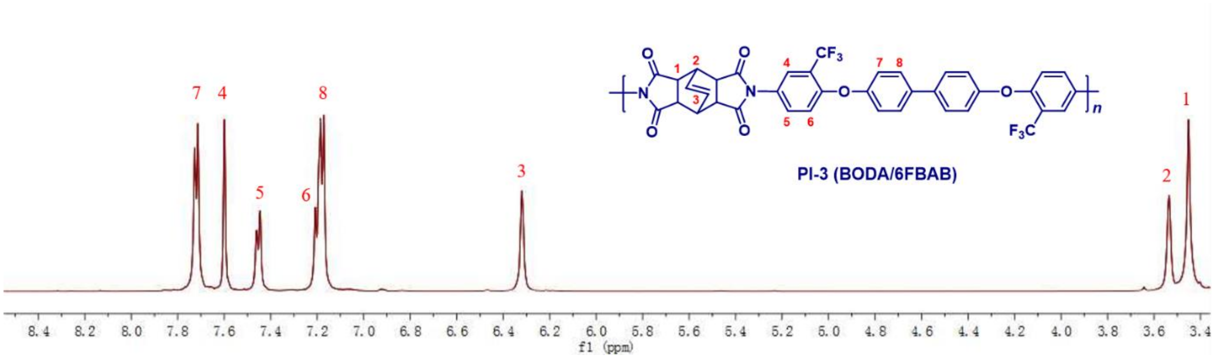

PI-3 (BODA/6FBAB)
f1 (ppm)

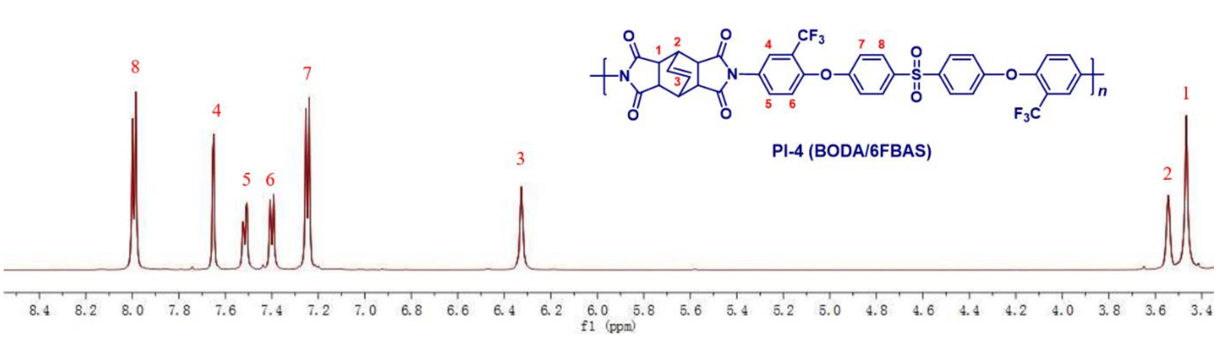

PI-4 (BODA/6FBAS)
f1 (ppm)

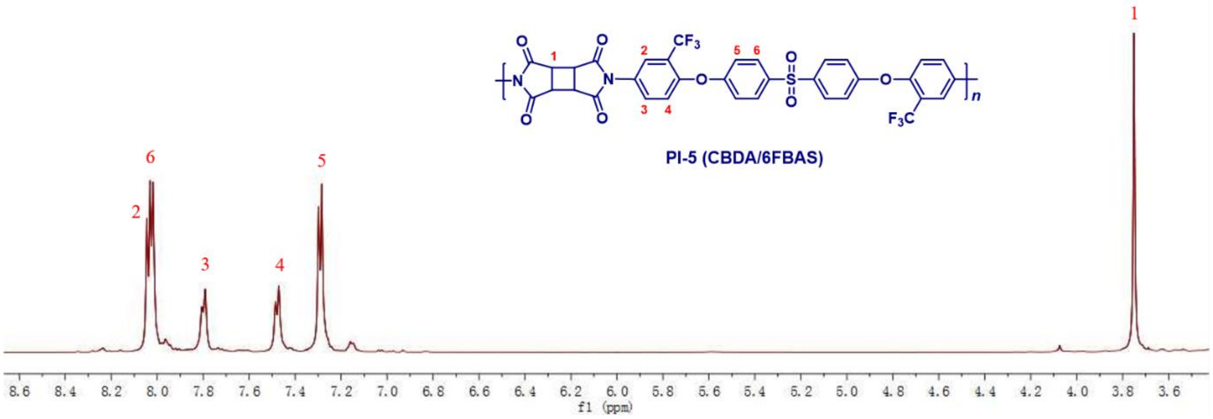

Figure S2. ^{1}H NMR spectra of the semi-alicyclic polyimides in DMSO-d_6.

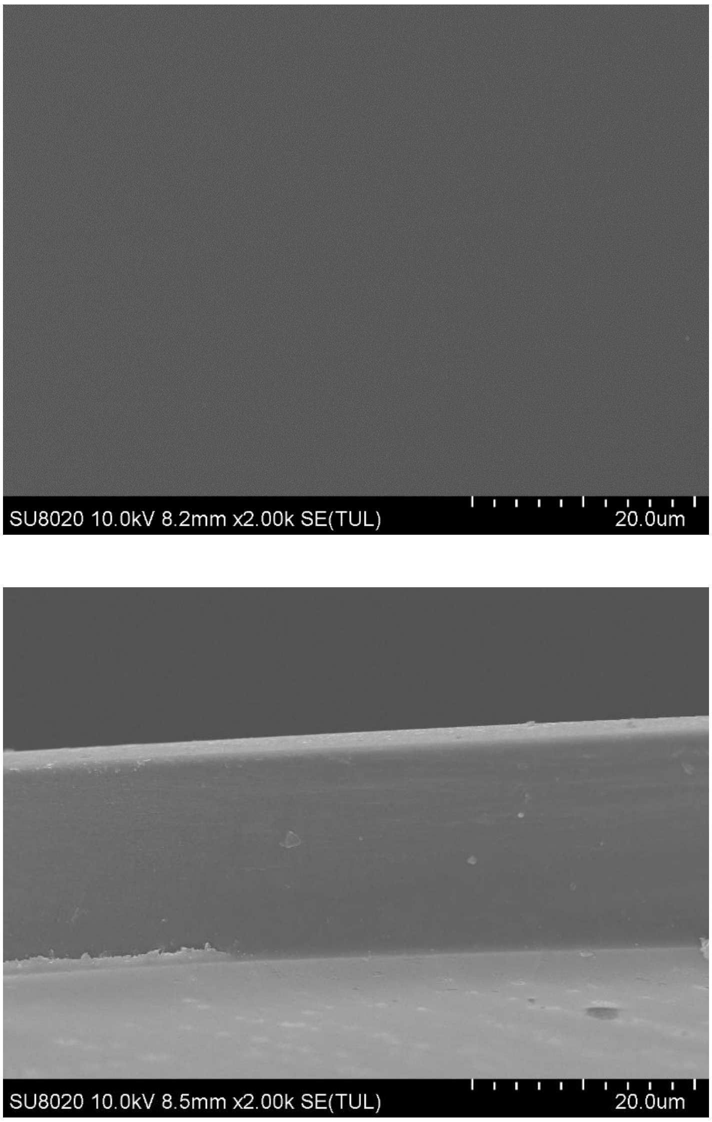

Figure S3. Scanning electron microscopy (SEM) images of the surface and cross-sectional fracture of PI-1 film.

SEM (*Hitachi SU8020, Japan*) was performed at an acceleration voltage of 10.0 kV.

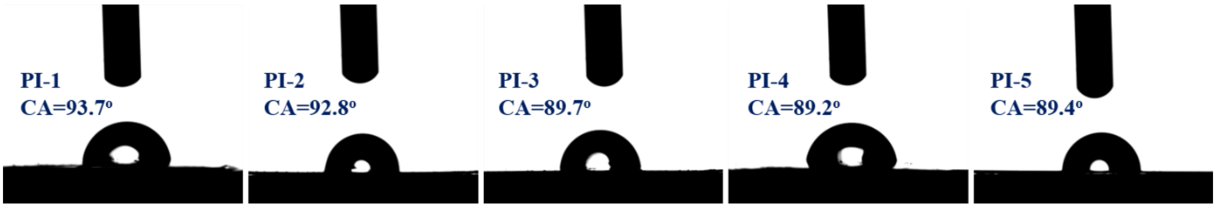

Figure S4. Water contact angle (CA) images of the semi-alicyclic polyimide films.

Static contact angle tests were performed with a Dataphysics OCA 25 optical contact angle system at room temperature. The surface of specimen was wiped with ethanol and dried before testing. Distilled water was used and at least three measurements were taken for each sample.

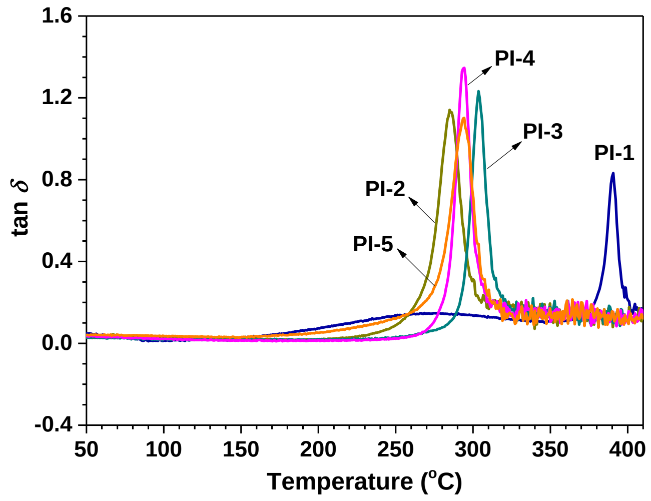

Figure S5. DMA curves of semi-alicyclic polyimide films in nitrogen.

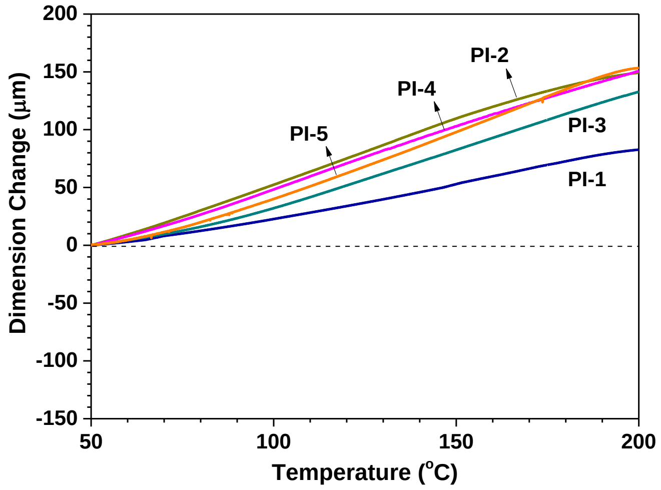

Figure S6. TMA curves of semi-alicyclic polyimide films in nitrogen.

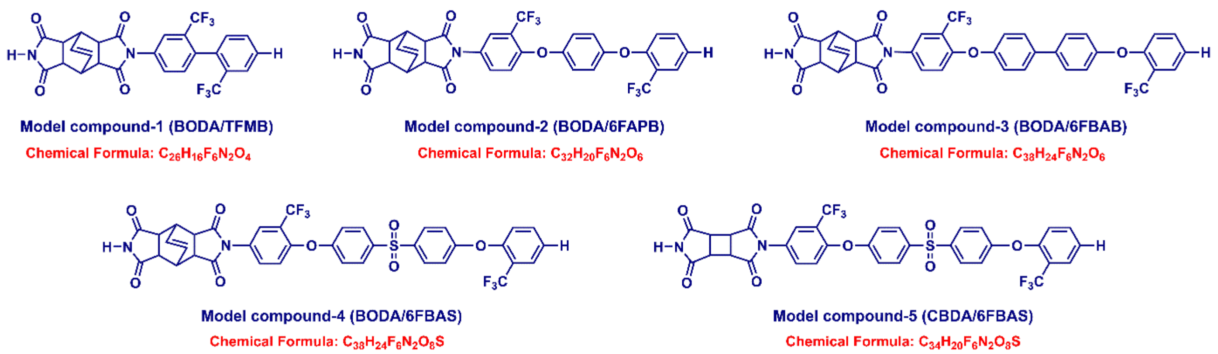

Figure S7. Model compound structures of semi-alicyclic polyimides for the DFT calculations.

Table S1. Representative structures and performance comparison of different semi-alicyclic polyimides prepared in the present and previous work*.

**Polyimides**	***Chemical Structures***	***Solubility***	***Chemical Resistance***	***Fluorine Content* (%)**	**W_A (%)**	**T_S (MPa)**	**E_B (%)**	**T_g (°C)**	**CTE (ppm/°C)**	ε_{opt}	**λ_0 (nm)**	**T_{400} (%)**	***b****	**E_{GAP} (eV)**
PI-1 (BODA/TFMB)	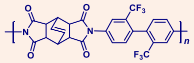	√	× / √	21.4	0.78	98	5.1	390	34	2.61	298	83	3.8	5.43
PI-2 (BODA/6FAPB)	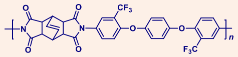	√	× / √	17.8	0.92	84	8.0	285	62	2.67	303	74	4.0	5.21
PI-3 (BODA/6FBAB)	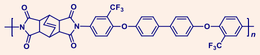	√	× / √	15.9	1.08	77	10.1	304	55	2.75	313	68	4.2	4.92
PI-4 (BODA/6FBAS)	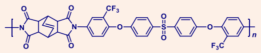	√	× / √	14.6	0.77	95	9.1	294	63	2.73	299	87	3.4	5.06
PI-5 (CBDA/6FBAS)	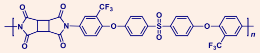	√	× / √	15.6	1.05	91	8.0	295	64	2.76	301	84	4.0	4.98
Ref-PI (BODA/BAPM)	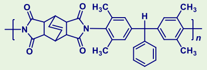	√	√ / √	0.0	2.67	76	5.4	>400	49	2.80	283	72	7.7	4.70
Ref-PI (BODA/BAFM)	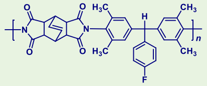	√	√ / √	3.4	2.51	81	6.1	387	47	2.77	283	72	7.1	4.75
Ref-PI (BODA/BATFM)	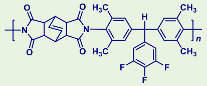	√	√ / √	9.6	1.53	91	6.4	364	52	2.73	282	80	6.8	4.94
Ref'-PI (HPMDA/TFMB)	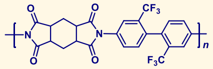	√	× / √	22.4	0.59	98	6.6	370	35	-	292	85	3.6	5.31
Ref'-PI (HPMDA/6FAPB)	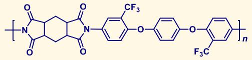	√	× / √	18.5	0.81	106	9.1	268	62	-	302	88	3.7	5.22
Ref'-PI (HPMDA/6FBAB)	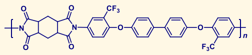	√	× / √	16.5	0.95	108	11.6	276	61	-	314	87	3.8	4.95

Ref'-PI (HPMDA/6FBAS)	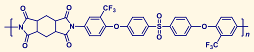	√	× / √	15.1	0.92	98	6.6	280	62	-	298	88	3.3	5.21
Fully-aromatic PI (PMDA/ODA, *Kapton*)	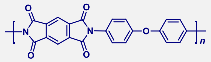	×	√ / √	0.0	2.65	–	–	417	0.0485	3.22	444	0	107.7	2.88

* Solubility: measured in the polar solvents including NMP, DMAc, DMF at 25 °C; Chemical resistance (MEK/KOH): evaluated by dipping film in methyl ethyl ketone (MEK) and potassium hydroxide solution (10 wt%) at 25 °C for 5 min, respectively.; Fluorine content: fluorine content in the repeating unit; W_A: water absorption rate at 25 °C for 24 h; T_S: average values of tensile strength; T_g: the glass-transition temperature; CTE: in-plane coefficient of thermal expansion, 50–200 °C for PI-1~PI-5 and Ref'-PI series, and 50–250 °C for Ref-PI series; ε_{opt}: optical dielectric constant estimated by average refractive index method, $\varepsilon_{opt} = 1.1n_{av}^2$; λ_0: UV cutoff wavelength; T_{400}: transmittance at 400 nm with film thickness of 25±3 μm; b^*: yellow color parameter calculated according to CIE LAB equation, measured with film thickness of 50±3 μm; E_{GAP}: energy band gap, $E_{GAP} = E_{LUMO} – E_{HOMO}$.
